# Does selective root canal retreatment preserve the tooth’s fracture resistance? An ex-vivo study

**DOI:** 10.1186/s12903-024-05002-1

**Published:** 2024-10-19

**Authors:** Mohammed Turky, Yasmine Ahmed Mortada Abd Elfatah, Shaimaa Hamdy

**Affiliations:** 1 Department of Endodontics, Faculty of Dentistry, Minia University, Minia, Egypt; 2 Department of Endodontics, Faculty of Dentistry, Sphinx University, Assiut, Egypt; 3 Department of Paediatric Dentistry and Dental Public Health, Faculty of Dentistry, Assiut University, Assiut, Egypt; 4 Department of Oral Diagnosis, Oral Medicine and Periodontology, Faculty of Dentistry, Nahda University, Beni Swef, Egypt

**Keywords:** Endodontics, Minimally invasive approach, Retreatment, Root canal therapy, Selective retreatment

## Abstract

**Objectives:**

To assess the tooth’s fracture resistance when submitted to a selective root canal retreatment compared to the conventional approach.

**Methods:**

33 intact permanent mandibular first molars were selected according to specific criteria. After teeth mounting, the primary root canal treatment was conducted and followed by thermo-mechanical aging procedures to mimic a few clinical conditions. The specimens were randomly divided into three groups (*n* = 11); a control group in which intact teeth were used and two experimental groups according to the retreatment approach: conventional non-surgical retreatment (Conventional-NSR), and selective non-surgical retreatment (Selective-NSR). Later, the teeth were submitted to a final thermo-mechanical aging procedure and tested regarding their fracture resistance (static fracture test). The maximum load to fracture was recorded as were the types of failure modes (repairable or non-repairable fracture). A proper statistical analysis was conducted, considering a significance level of 5%.

**Results:**

The Conventional-NSR group showed a mean failure load of 867.7 ± 108.9 N while the Selective-NSR group had 1106.8 ± 159.8 N (*P* = 0.012). Both retreatment groups showed significantly lower results when compared to the control group. Additionally, the Conventional-NSR group showed higher proportions of non-repairable fractures (54.5%) when compared to both the Selective-NSR (36.4%) and control (18.2%) groups.

**Conclusions:**

Selective root canal retreatment preserved the tooth’s fracture resistance compared to the conventional retreatment approach.

**Clinical trial number:**

Non-applicable. Conducting the current experiment was limited to obtaining approval from the local Research Ethics Committee at the Faculty of Dentistry, Minia University (Committee No. 105, Registration No. 902, Date: 26/3/2024).

**Supplementary Information:**

The online version contains supplementary material available at 10.1186/s12903-024-05002-1.

## Introduction

The primary goal of root canal treatment is to treat or prevent apical periodontitis [1]. Despite the predictability of root canal treatment in achieving this goal [2], failure in the form of newly emerging, persistent, or recurrent post-treatment diseases can occur [3–5]. Therefore, a substantial need for further intervention may be required in many instances.

Given the complexities of root canal anatomy, particularly in multi-rooted molar teeth that may hinder effective disinfection, the possibility of missed anatomy [6], and the significant role intra-radicular bacteria play in endodontic failure [7–9], conventional non-surgical root canal retreatment can be preferred over surgical approaches [10, 11]. Nevertheless, it has been reported that secondary root canal treatment reduces the tooth’s fracture resistance [12, 13].

Selective root canal retreatment is a newly emerging concept, first described in the scientific literature by Nudera in 2015, as a viable option for the retreatment of failed root canal-treated teeth with a periapical lesion involving one root while the other(s) is/are not affected [14]. Like any approach, there are benefits and drawbacks to this novel strategy. On the positive side, it involves more conservative access to the affected root, preserving more tooth structure as well as direct or indirect restorations. Additionally, it may reduce the incidence of iatrogenic errors and the costs of the treatment, since only the affected root is treated. The major drawback is the possibility of the development of new periapical involvement around the untreated root(s). Recent publications confirmed favorable outcomes and reasonable survival rates achieved by this conservative approach [15, 16]. Moreover, another recent study based on the Dutch Healthcare system pointed out the possibility of the cost-effectiveness of this technique [17]. Notwithstanding all these temptations, the selective retreatment method has not yet been evaluated from a mechanical standpoint, which could be the primary factor for the emergence of such a minimally invasive approach. Perceiving the significance of the mechanical perspective and its pivotal effect on the longevity and retention of endodontically treated teeth [18], the present study sought to investigate the mechanical performance of root canal-filled teeth retreated with such a novel concept. Therefore, the present study aimed to compare the impact of the selective root canal retreatment to the conventional retreatment on the fracture strength of endodontically retreated teeth.

The null hypothesis tested was that no significant difference in tooth fracture resistance would be observed between selective and conventional non-surgical root canal retreatment techniques.

## Methods

### Ethical regulation

The current investigation was carried out after receiving approval from the Research Ethics Committee of the University where the study was performed (Committee No. 105, Registration No. 902, Date: 26/3/2024).

### Sample size calculation

Based on the results of a pilot study conducted on ten teeth in each group, the sample size was calculated, and the effect size (f) for the One-Way ANOVA test was 0.7 using an alpha (α) level of 0.05 and a beta (β) level of 0.95 (i.e., power = 95%). The total sample size was 33 teeth (11 per group). Sample size calculation was performed using G* software, version 3.1.9.7(G*Power 3.1.9.7, Heinrich Hein University, Düsseldorf, Germany).

### Criteria for sample selection (strict criteria)

A total of 33 freshly extracted intact human permanent mandibular first molars, extracted for periodontal reasons with minimal trauma (atraumatic extraction), and fully developed two roots, were collected from the outpatient clinic at the Faculty of Dentistry, Minia University, Egypt. The mesial root had a Vertucci’s type IV root canal system, while the root canal configuration in the distal root was a Vertucci’s type I [19]. All selected teeth exhibited equivalent root and root canal curvature angles, less than 20 degrees, as determined by Schneider’s method [20], and a radius of less than 5 mm. Pair matching for tooth morphology, dimensions, pulp morphology, and volume was achieved using 3D imaging (Papaya 3D plus, Genoray, Gyeonggi-do, Korea). Tooth dimensions were measured in the buccolingual and mesiodistal directions using a digital caliper with an accuracy of 0.01 mm. The buccolingual dimension of the selected teeth was 7 mm ± 0.5 mm, while the mesiodistal dimension was 9 mm ± 0.5 mm. To standardize tooth length, the occlusal table was reduced to 20 mm. Teeth with caries, un-negotiable canals, resorptive lesions (external or internal), and pre-existing restorations or root canal fillings were excluded. Additionally, teeth were inspected for signs of cracks or fractures at 20X magnification using a dental operating microscope (DOM) (Magna Labomed, Labo America Inc., 920 Auburn Court Fremont, CA 94538, USA). The selected teeth were ultrasonically cleaned to remove any attached hard or soft deposits, disinfected in a full concentration (5.25%) sodium hypochlorite solution (NaOCl) (Omez, Phar Omez, Pharaonic Pharmaceuticals, Egypt) for 30 min, and finally immersed in a 0.1% thymol suspension (Formula e Acao, São Paulo, SP, Brazil) until use.

### Teeth mounting

**Fig. 1:**
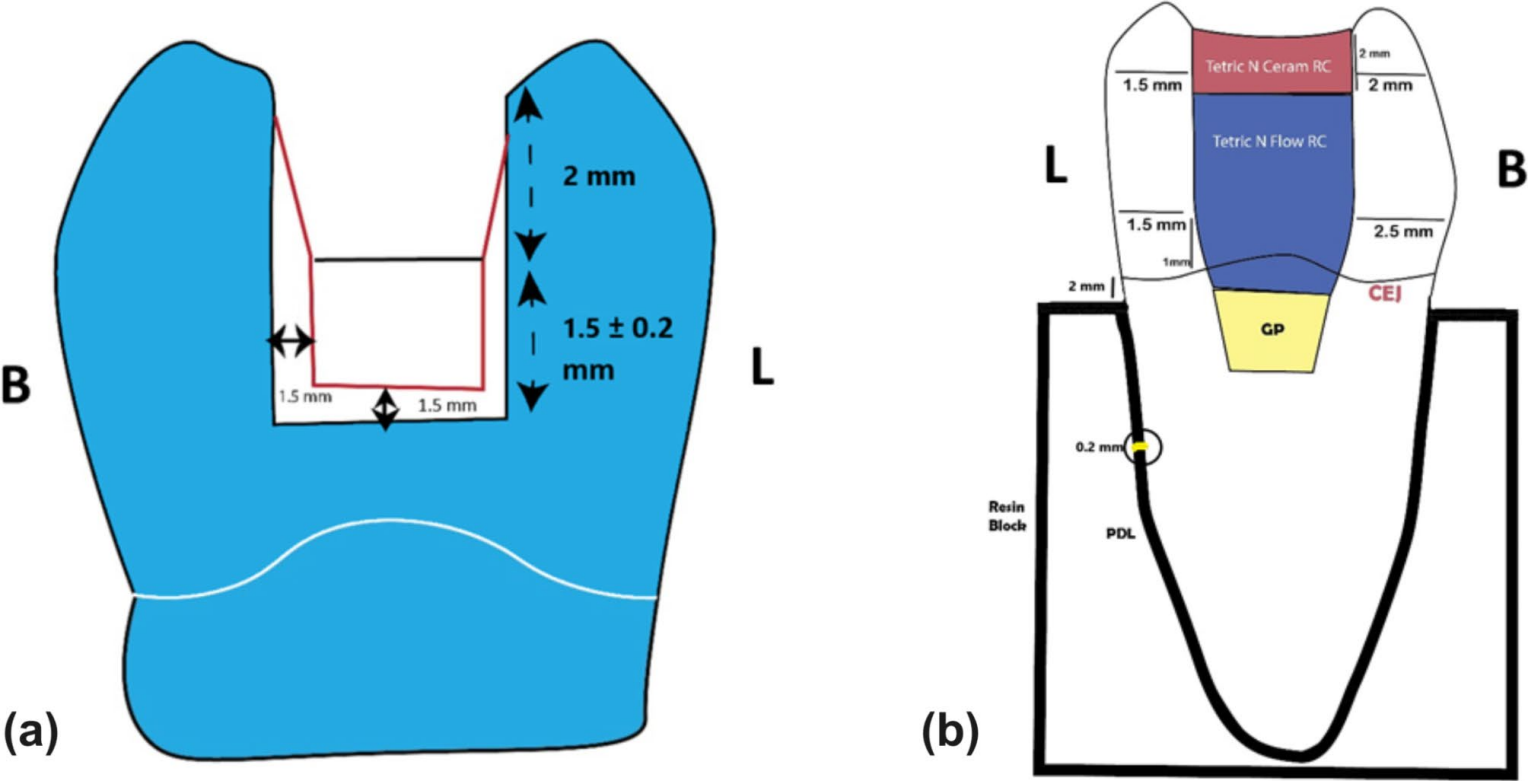
Illustrations of the dimensions of the initially prepared cavity (**a**) and of the post-endodontic restoration (**b**)

Two Teflon half-split molds with a diameter of 30 mm and a height of 25 mm were used. The roots of each tooth were covered with a stretch film with a thickness of 0.2 mm. After pouring a self-curing resin into the mold, each tooth was immersed in the acrylic resin up to 2 mm apical to the cementoenamel junction (CEJ) to simulate the bone level [21]. To ensure correct centering and alignment parallel to the tooth’s long axis, a Dental Surveyor (Ney Dental Surveyor, Anaheim, CA, USA) was used to position each tooth inside the acrylic block. After the acrylic resin had set, the teeth with the stretch film were removed, and light body silicone (Elite HD; Zhermack SpA, Badia Polesine, Italy) was injected into the root cavity to replicate the periodontal ligament [21, 22]. Any excess impression material was removed using a #12 scalpel. Mesio-occluso-distal (MOD) cavities were then prepared using a carbide bur (straight round end fissure #245; Kerr, Kloten, Switzerland) in a high-speed handpiece (Sirona, Erlangen, Germany) with water cooling. The measurements of 2 ± 0.2 mm pulpal depth, 1.5 ± 0.2 mm axial height, 1.5 ± 0.2 mm axial depth, and 1.5 ± 0.2 mm gingival width were used to determine the occlusal isthmus width, set at one-third of the intercuspal distance (Fig. 1a) [23].

### Primary endodontic treatment

All endodontic and restorative procedures, including the access cavity design, root canal instrumentation, irrigation protocol, root canal filling, and coronal restoration, were identical in all experimental samples and performed by a single experienced operator with 14 years of experience in endodontics (Y.A.M.). A traditional access cavity was designed following pre-established guidelines for access cavity preparation to achieve straight-line access to the canal orifices with smoothly divergent cavity walls [24]. Strictly adhering to those guidelines, the entire roof of the pulp chamber was removed using a round diamond bur #801 − 014 (Komet Den Komet Dental, Braseler GmbH & Co. KG, Lemgo, Germany), and to enhance visibility and accessibility, the cavity walls were refined using a tapered carbide fissure bur (Komet H33L, Komet Den Komet Dental, Braseler GmbH & Co. KG, Lemgo, Germany). All burs used for access cavity preparation were mounted on a high-speed handpiece with water spray. According to a previous study by Garlapati et al. [25], the access cavities were created while preserving the following wall thicknesses: the lingual wall measured 1.5 mm at both the gingival floor level and the occlusal surface, and the buccal wall measured 2 mm at the occlusal surface and 2.5 mm at the gingival floor level (Fig. 1b). Using a digital caliper, the widths of the remaining wall thickness were measured. After gaining access to the root canal orifices, patency was checked with a manual stainless-steel K-file ISO size 0.10 (Dentsply, Maillefer, Ballaigues, Switzerland); otherwise, the teeth with non-negotiable root canals were discarded and replaced. Root canals in all samples were standardized to have initial apical diameters equivalent to ISO size 0.15 for mesial canals, while the initial apical diameters of the distal canals were equivalent to ISO size 0.25. Subsequently, the working length was visually determined through the insertion of a manual stainless-steel K-file ISO size 10 until visible beyond the apical foramen, then 1 mm was subtracted from this measurement, and a manual glide path was established using a hand stainless-steel K-file ISO size 0.15 (Dentsply, Maillefer, Ballaigues, Switzerland). For more accurate standardization of the working length, definite reference points were determined for each canal but were similar in all samples. Root canal instrumentation was accomplished using a crown-down technique with Hyflex CM rotary files (Hyflex CM, Coltene Whaledent, Cuyahoga Falls, OH, USA) mounted on a controlled torque electrical endodontic motor (TriAuto mini; J. Morita MFG, CORP, Japan). The files were used in a continuous rotation motion after adjusting the speed and torque according to the manufacturer’s instructions. The mesial root canals (mesiobuccal and mesiolingual) were prepared up to 0.30/0.04, while the distal canal was instrumented up to 0.40/0.04, with 2 ml of 5.25% NaOCl irrigation for 20 s performed between files. A final rinse was performed in the following sequence: 10 ml of 5.25% NaOCl for 2 min followed by 10 ml of 17% ethylenediaminetetraacetic acid (EDTA) solution for 2 min, with the same volumes of saline in between and as a final flush to eliminate any irrigant residues. After the chemo-mechanical preparation, the root canals were dried using matched paper points (Coltene Whaledent, Cuyahoga Falls, OH, USA) and filled using a single-cone technique with matched master gutta-percha cones (Coltene Whaledent, Cuyahoga Falls, OH, USA) and a bioceramic sealer (EndoSequence BC sealer, Brasseler Blvd, Savannah, USA). The coronal level of the obturation was standardized to be 1 mm below the canal orifices. Any remaining sealer residue was removed from the pulp chamber using a cotton pellet saturated with 70% alcohol [26, 27]. The quality of root canal filling was ensured using conventional periapical radiographs. Finally, the access cavity was sealed with bulk-fill flowable resin composite (Tetric N-Flow Bulk Fill, Ivoclar Vivadent Zurich, Switzerland), filling most of the access cavity and leaving 2 mm for the final resin composite restoration (Tetric N-Ceram, Ivoclar Vivadent Zurich, Switzerland) (Fig. 1b).

### Thermo-mechanical cycling (aging procedures)

The mechanical aging test was conducted using a four-station multi-modal ROBOTA chewing simulator (ACTA Fatigue tester, Amsterdam, Netherlands). The simulator had servomotor control (Model Ach-09075dc-T, Ad-Tech Technology, Berlin, Germany) and a thermo-cyclic protocol, which simulated simultaneous motions under thermodynamic circumstances in both vertical and horizontal directions. Each chamber had a lower plastic sample holder where the specimen could be implanted, and an upper hardened steel stylus holder that could be tightened to act as an antagonistic substance. For the chewing simulation, a weight of five kilograms (49 N) was applied. The following parameters were used: 1.6 Hz cycle frequency, 90 mm/s rising/forward speed, 40 mm/s descending/backward speed, and 1 mm horizontal movement. To simulate six months of intraoral aging, the specimens underwent 75,000 cycles with 600 thermal cycles (5˚/55˚C, dwell length of 25 s) [28].

### Specimens grouping and retreatment procedures

Teeth were numbered and randomly distributed into two groups according to the retreatment approach employed. The first group, labeled as the conventional non-surgical retreatment group (Conventional-NSR) (*n* = 11), involved the retreatment of all root canals after gaining a traditional access cavity to the root canal orifices. The second group, referred to as the selective non-surgical retreatment group (Selective-NSR) (*n* = 11), underwent retreatment restricted to the distal root canal, with an access opening limited to the distal canal only (an orifice-directed access). Additionally, 11 intact mandibular first molars were used as controls.

**Fig. 2:**
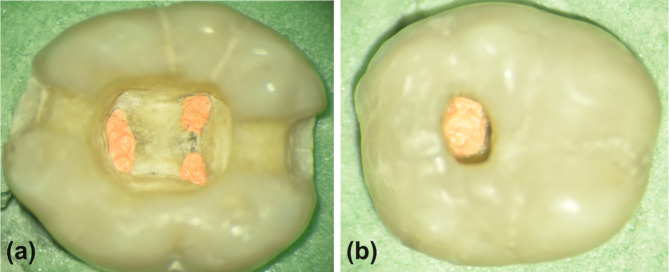
Representative images of the traditional access cavity for conventional non-surgical retreatment (**a**) and of the orifice-directed access for selective non-surgical retreatment (**b**)

In the Conventional-NSR group (Fig. 2a), a traditional access cavity was established, as previously described, after removing the entire coronal restoration. The root canal filling was removed using the same engine-driven file system (Hyflex CM) that was used during the primary root canal preparation, with matched sizes of instruments used for each root canal. Between files, 2 ml of 5.25% NaOCl irrigation for 20 s was delivered, and the final rinse protocol was accomplished in the same manner as in the primary root canal treatment. Retreatment procedures ceased when no filling remnants were visible along the shaft of a hand stainless steel H file (Dentsply, Maillefer, Ballaigues, Switzerland), corresponding to the size of the master apical file for each root canal, and a confirmatory conventional periapical radiograph was taken. Following this, the root canal preparation, obturation, and final coronal restoration were repeated as described previously.

In the Selective-NSR group (Fig. 2b), a conservative access cavity directed to the distal root canal was performed through the pre-existing coronal restoration under 16X magnification using a dental operating microscope (DOM). This orifice-directed access was prepared utilizing a diamond round bur #806 − 010 (Komet Dental, Braseler GmbH & Co. KG, Lemgo, Germany), mounted on a high-speed handpiece with water spray, and standardized in all samples. It left 2 mm ± 0.5 mm of the restoration/tooth both buccally and palatally, while 5 mm ± 0.5 mm and 2 mm ± 0.5 mm of the restoration remained mesial and distal to the access opening, respectively. The root canal obturation in the distal canal was removed using Hyflex CM rotary files up to size 0.40/0.04, with 2 ml of 5.25% NaOCl irrigation for 20 s utilized between files with the same final rinse as previously mentioned. The retreatment procedure was terminated when filling residues were no longer evident along the shaft of a hand stainless steel H file ISO size 0.40, verified by a conventional periapical radiograph. Subsequently, the previously mentioned procedures for root canal preparation and obturation were repeated. Finally, the access opening was sealed with bulk-fill flowable resin composite (Tetric N-Flow Bulk Fill), filling the majority of the access cavity and leaving 2 mm for the final resin composite restoration (Tetric N-Ceram).

All retreatment procedures were performed by another experienced endodontist, M.T., who has 20 years of experience in endodontics.

### Final thermo-mechanical cycling (final aging procedures)

All teeth (in the control and experimental groups) were aged to the equivalent of six months of intraoral aging using the Robota chewing simulator for an additional 75,000 cycles, along with 600 thermal cycles (5˚/55˚C, with a dwell period of 25 s).

### Fracture test (static fracture test)

Each sample was individually mounted on a materials testing apparatus (Model 3345; Instron Industrial Products, Norwood, MA, USA), which was computer-controlled and equipped with a 5 kN load cell. Data were collected using computer software (Instron^®^ Bluehill Lite Software). Screws were tightened to securely attach the samples to the lower fixed compartment of the testing machine. To conduct the fracture test, a metallic rod with a round tip (7.8 mm diameter) was affixed to the upper movable compartment of the testing machine. This rod traveled at a cross-head speed of 1 mm/min and applied compressive load occlusally in a direction parallel to the long axis of the root. The load-deflection curve was recorded using computer software (Bluehill Lite Software, Instron^®^ Instruments). The occurrence of an audible crack confirmed the load at failure. The load required to cause fracture was measured in newtons.

### Failure mode assessment

**Fig. 3:**
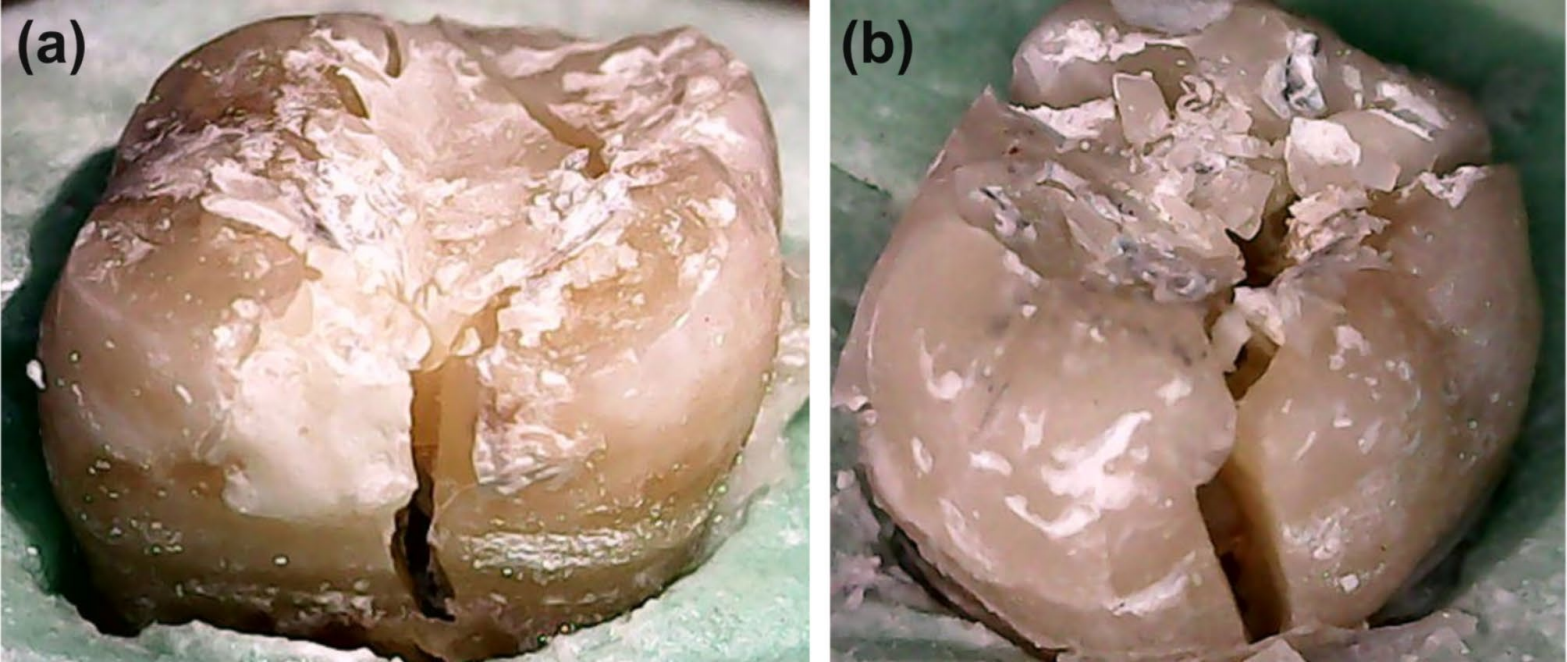
Examples of repairable fracture (**a**) and non-repairable fracture (**b**)

A USB digital microscope (U500x Digital Microscope, Guangdong, China) with 35X magnification was utilized to assess failure patterns. Images were captured and transferred to a personal computer (PC) equipped with image analysis software (Image J 1.43U, National Institute of Health, USA) to determine the failure mode pattern and whether the fracture was unrestorable/non-repairable (i.e., fracture occurring more than 1 mm below CEJ) or restorable/repairable (i.e., fracture occurring 1 mm above CEJ) (Fig. 3) [29].

The images were captured using a digital camera (U500x Digital Microscope, Guangdong, China) with a resolution of 3 megapixels, positioned vertically at a distance of 2.5 cm from the samples, with the lens axis at an angle of approximately 90° to the light sources. Illumination was provided by 8 LED lamps with a color index close to 95%. Images were taken at a fixed magnification of 35X and maximum resolution before being transferred to a PC. The resolution at which each image was captured was 1280 × 1024 pixels.

### Statistical analysis

The numerical data, expressed as mean and standard deviation values, underwent normality testing using Shapiro-Wilk’s test (0.118). Subsequently, and due to the Gaussian distribution of the outcomes, one-way ANOVA was employed to compare the mean changes among the three groups. A significance threshold of *P* < 0.05 was utilized to ascertain statistically significant differences.

## Results

**Fig. 4:**
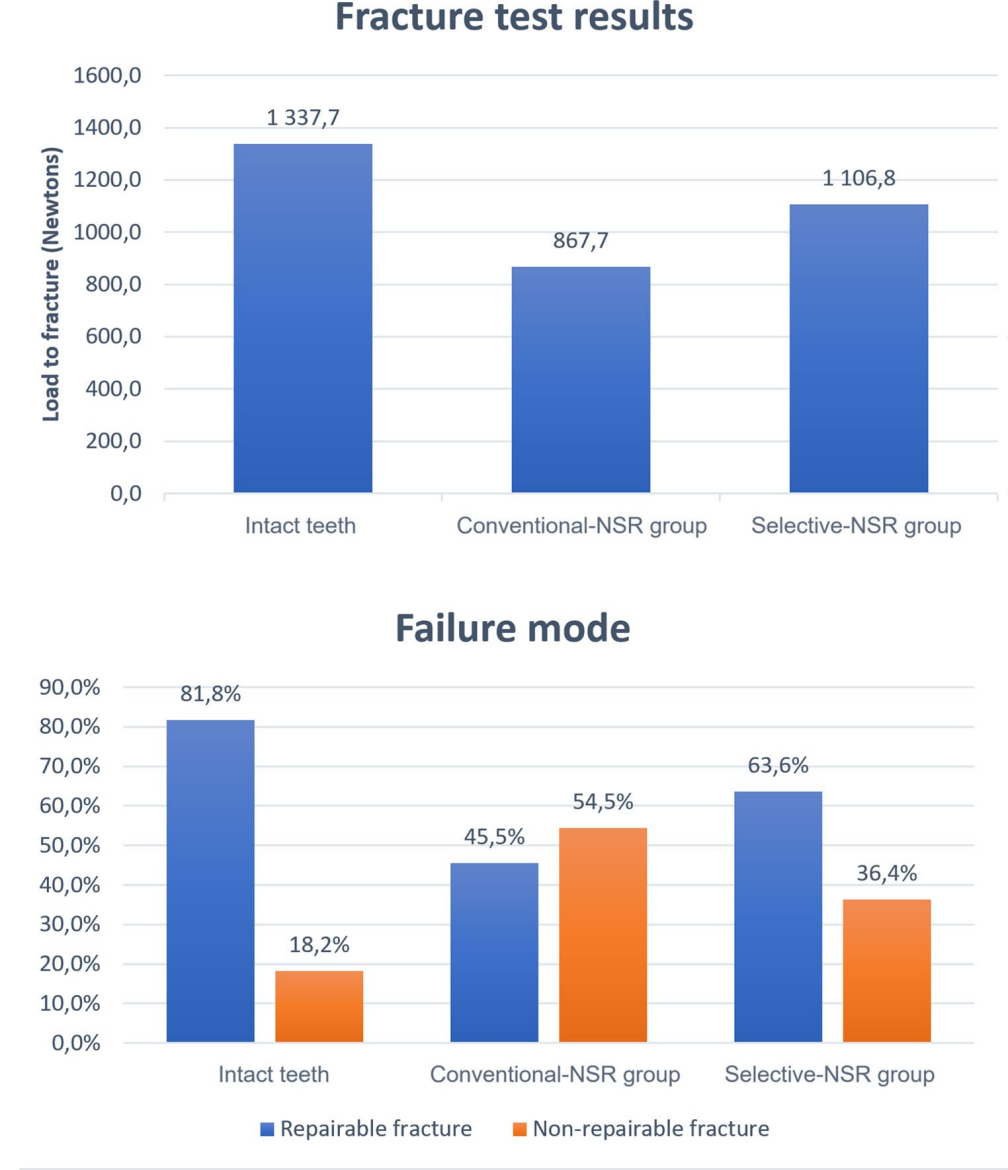
Results for the fracture test (top) and failure mode (bottom) assessments

The fracture test results showed a statistically significant difference between the Conventional-NSR group (mean failure load of 867.7 ± 108.9 N) and the Selective-NSR group (1106.8 ± 159.8 N) (*P* = 0.012), favoring the latter. Both NSR groups presented significantly lower fracture resistance when compared to the control group (1337.7 ± 250.5 N) (Table 1; Fig. 4). Regarding the failure mode, the Conventional-NSR group tended to present higher proportions of non-repairable fractures (54.5%), while both the Selective-NSR (36.4%) and control (18.2%) groups tended to show the opposite (Table 1; Fig. 4).

**Table 1:** Fracture test and failure mode assessment results

Group	*n*	Fracture test	Failure mode (fracture type %)	
		(Newtons)	Repairable	Non-Repairable
Intact teeth	11	1337.7 ± 250.5^a^	81.8%	18.2%
Conventional-NSR	11	867.7 ± 108.9^b^	45.5%	54.5%
Selective-NSR	11	1106.8 ± 159.8^c^	63.6%	36.4%

Different superscript letters represent statistically significant differences within that column (*P* < 0.05)

## Discussion

It has been demonstrated that one-third of root canal-treated teeth may fail even with proper treatment [30–32]. Endodontic failure can be either biological or mechanical [33]. Mechanical failure can lead to disastrous consequences, resulting in the loss of root canal-treated teeth [34], whereas biological failure may afford the tooth another chance at survival if properly retreated [10, 11]. Retreatment of failed root canal-treated teeth can be achieved through either conventional non-surgical root canal retreatment or surgical procedures [35]. Although both approaches can be successful when correctly applied, conventional non-surgical retreatment can be the preferred choice if access to the root canals is available [35, 36]. This involves the removal of root canal filling from all root canals after obtaining adequate access to the root canal orifices to reassess root canal preparation and disinfection [37]. However, a recent systematic review and meta-analysis have indicated that conventional root canal retreatment may compromise the tooth’s fracture resistance [13].

Selective retreatment is a minimally invasive approach for root canal retreatment, involving the retreatment of the root canals associated with a periapical lesion after establishing an access cavity directed to the orifices [14, 15]. Despite the predictability of this approach [14–16], it remains unclear whether such a minimally invasive strategy can sustain the tooth’s fracture resistance. Therefore, the present study aimed to compare the impact of the selective retreatment approach with traditional non-surgical retreatment on the fracture resistance of the tooth.

Mandibular first molars are commonly affected by carious lesions due to their early emergence in the oral cavity and typically require endodontic treatment [38]. It has been observed that MOD cavities in root canal-treated teeth increase their vulnerability to fracture [39]. Recent research has shown that root canal-treated molars restored with direct restorations are most frequently susceptible to root canal retreatment [40]. Therefore, mandibular first molars with MOD cavities restored with direct restorations were selected for the present study. It has been reported that the distal root has a higher frequency of missed canals and, consequently, a higher incidence of periapical pathosis compared to the mesial root [41]. Additionally, the complexity of the root canal anatomy and the possibility of encountering buccolingual curvature in the mesial root make the distal root easier to standardize in the ex vivo environment [42]. All these factors contributed to selecting the distal root for retreatment in the Selective-NSR group.

In an attempt to mitigate the known risk of bias in fracture tests [43], sample selection and preparation were subjected to strict criteria. Only freshly extracted intact teeth, extracted with minimal trauma, were selected. They were standardized using 3D imaging to ensure uniformity in dimensions, morphology, pulp space morphology, and volume. These teeth were then stored wet in a preservative medium until they were used. Subsequently, all samples underwent identical endodontic and restorative procedures, ensuring standardization of root canal preparation dimensions, access cavities, and MOD cavities.

It has been demonstrated that fracture tests are significantly impacted by the root embedment procedure and the simulation of the periodontal ligament [44]. Furthermore, tooth age is one of the overlooked aspects in most fracture resistance investigations, which has been found to negatively affect the tooth’s fracture strength [45, 46]. Taking these considerations into account, and to replicate oral circumstances, all teeth were collected from a population between 20 and 30 years old and embedded into a silicone impression material surrounded by acrylic resin up to 2 mm apical to CEJ to simulate the periodontal structure and the bone level, respectively [21, 22], and subjected to thermo-mechanical fatigue equivalent to 6 months of intra-oral aging [28].

Fracture resistance could be tested using static and dynamic methods. While dynamic tests simulate the clinical situation, static testing is the most widely used and effective method, as variations in dynamic testing make it difficult to correlate results [47]. Therefore, static fracture testing was employed in the current study.

The present study revealed that both retreatment approaches significantly reduced the resistance of the teeth to fracture. Among other aspects involved in root canal treatment/retreatment, research has linked the reduction in mechanical performance to tooth structure loss and defects produced by caries removal, access cavity creation, and manual or rotary instrumentation [12, 13, 48] .

When comparing the selective retreatment approach to conventional root canal retreatment, the present findings indicate that the selective retreatment approach significantly preserves the fracture resistance of the root canal-retreated teeth more than its conventional counterpart, with more repairable fracture patterns. Thus, the null hypothesis was rejected. This might be attributed to the more extensive interventional process associated with the conventional retreatment approach, which involved the total removal of the entire coronal restoration followed by the retreatment of all the root canals. It has been evident that the complete removal of the pre-existing coronal restoration is associated with the additional removal of surrounding tooth substance, resulting in subsequent weakening of the remaining structure and a larger restoration that performs less efficiently than a smaller one [49]. Additionally, polymerization shrinkage stresses resulting from the replacement of a new resin composite may lead to a fracture of the adjacent tooth substance [49]. Research has shown that root canal retreatment might be associated with more defects than those produced by the primary endodontic treatment, and consequently, the mechanical properties of the tooth may be more negatively influenced [12].

The current investigation suggests that selective retreatment may be a promising approach for preserving the fracture resistance of endodontically treated multi-rooted teeth.

Since clinical testing of the subjects’ fracture resistance is not feasible, this experiment was conducted ex vivo. While the major limitation of the current study lies in its ex vivo design, ex vivo investigations are considered a double-edged sword. Despite offering a lower level of evidence, these types of studies allow for the standardization of all variables that are impossible to standardize in a clinical setup. Moreover, all factors that may threaten the validity of the results were considered during the setting of the present study, which may lead to superior internal validity of the study.

As the current study focused on the mechanical aspects, complementing the limited available data on the biological predictability of this minimally invasive approach, further well-designed prospective randomized clinical trials are needed to monitor clinical and radiographic outcomes. Such research is essential to elevate the level of evidence supporting this approach and to confirm its applicability in routine clinical practice. Additionally, survival studies comparing the longevity and retention of teeth treated with selective root canal retreatment versus traditional non-surgical retreatment are necessary to further validate this method. Furthermore, future ex vivo or in vivo studies are needed to assess the impact of such a minimally invasive retreatment approach on the efficacy of removing the root canal filling and the quality of subsequent endodontic and restorative procedures.

## Conclusions

Despite the limitations of this ex vivo study, from a mechanical perspective, selective retreatment emerges as a promising alternative to conventional non-surgical root canal retreatment. It offers the potential to preserve the fracture resistance of failed endodontically treated multi-rooted teeth.

## Electronic supplementary material

Below is the link to the electronic supplementary material.

Supplementary Material 1

## Data Availability

Availability of the data and materials: All data or materials generated or analyzed during this study are included in this article.
