# Supplementary material for: Does selective root canal retreatment preserve the tooth’s fracture resistance? An ex-vivo study
Source: BMC Oral Health. 2024 Oct 19;24:1251. doi: 10.1186/s12903-024-05002-1 (PMC11490081; doi:10.1186/s12903-024-05002-1)
Supplement: Supplementary file 1 — Supplementary Material 1 [file 12903_2024_5002_MOESM1_ESM.docx]

**Table 1: Pair-matching of the volumes for each sample**.

Group 1 Group 2 Group 3

23.08 mm^3^  21.97 mm^3^ 24.93 mm^3^

24.91 mm^3^ 25.48 mm^3^ 25.09 mm^3^

28.42 mm^3^  27.26 mm^3^ 29.08 mm^3^

29.51 mm^3^ 30.14 mm^3^ 29.61 mm^3^

30.92 mm^3^  32.24 mm^3^ 31.95 mm^3^

31.02 mm^3^ 33.12 mm^3^ 33.9 mm^3^

34.79 mm^3^ 35.03 mm^3^ 34.57 mm^3^

36.02 mm^3^  37.52 mm^3^ 36.29 mm^3^

38.02 mm^3^ 39.55 mm^3^ 38.55 mm^3^

41.39 mm^3^  40.91 mm^3^ 40.01mm^3^

43.27 mm^3^ 44.01 mm^3^ 45.11 mm^3^
